# Effects of Non-Differential Exposure Misclassification on False Conclusions in Hypothesis-Generating Studies

**DOI:** 10.3390/ijerph111010951

**Published:** 2014-10-21

**Authors:** Igor Burstyn, Yunwen Yang, A. Robert Schnatter

**Affiliations:** 1 Department of Environmental and Occupational Health, School of Public Health, Drexel University, Nesbitt Hall, 3215 Market Street, PA 19104, USA; 2 Department of Epidemiology and Biostatistics, School of Public Health, Drexel University, Nesbitt Hall, 3215 Market Street, PA 19104, USA; E-Mail: yunwen.yang@drexel.edu; 3 Occupational and Public Health Division, ExxonMobil Biomedical Sciences Inc., 1545 U.S. Highway 22 East, Annandale, NJ 08801, USA; E-Mail: a.r.schnatter@exxonmobil.com

**Keywords:** false positive, false negative, Monte-Carlo simulation, study design, ase-control studies, measurement error, exposure misclassification, Bayesian, hypothesis-testing, power

## Abstract

Despite the theoretical success of obviating the need for hypothesis-generating studies, they live on in epidemiological practice. Cole asserted that “… there is boundless number of hypotheses that could be generated, nearly all of them wrong” and urged us to focus on evaluating “credibility of hypothesis”. Adopting a Bayesian approach, we put this elegant logic into quantitative terms at the study planning stage for studies where the prior belief in the null hypothesis is high (*i.e.*, “hypothesis-generating” studies). We consider not only type I and II errors (as is customary) but also the probabilities of false positive and negative results, taking into account typical imperfections in the data. We concentrate on a common source of imperfection in the data: non-differential misclassification of binary exposure classifier. In context of an unmatched case-control study, we demonstrate—both theoretically and via simulations—that although non-differential exposure misclassification is expected to attenuate real effect estimates, leading to the loss of ability to detect true effects, there is also a concurrent increase in false positives. Unfortunately, most investigators interpret their findings from such work as being biased towards the null rather than considering that they are no less likely to be false signals. The likelihood of false positives dwarfed the false negative rate under a wide range of studied settings. We suggest that instead of investing energy into understanding credibility of dubious hypotheses, applied disciplines such as epidemiology, should instead focus attention on understanding consequences of pursuing specific hypotheses, while accounting for the probability that the observed “statistically significant” association may be qualitatively spurious.

## 1. Introduction

Despite valiant attempts and theoretical success of eliminating hypothesis-generating studies by generating all possible hypotheses [1], such studies live on in epidemiological thinking and practice.The 23 September 2014 search in *PubMed* for terms “hypothesis-generating (Title)” retrieved 77 records with seven published in 2014 alone. When the term is not restricted to title, 896 records resulted. The practice of declaring to be openly engaging in hypothesis-generating studies does not exist on the fringes of academia but is still a mainstream practice, as exemplified in a 2011 article by a U.S. government epidemiologist that used NHANES data Schreinemachers [2] and a 2013 article by a clinical epidemiologists [3]. Cole asserted that “… there is boundless number of hypotheses that could be generated, nearly all of them wrong” and urged us to focus on evaluating “credibility of hypothesis” [1]. We shall attempt to put this elegant logic into quantitative terms, hoping that quantitative expression of ideas presented in [1] will help investigators account for the output of Cole’s hypothesis-generating machine to evaluate the credibility of their chosen hypothesis in terms of likelihood of any causal effect existing at all. We neither disparage nor promote the so-called “hypothesis-generating” studies but simply accept that in the minds of many researchers they persist. In fact, all our arguments hold for any study where it is known *a priori* that the chance of finding true signal above noise is small (e.g., exploratory analyses, surveillance, *etc*.). The method we put forward below is equally valid for understanding whether anything stands to be gained from conducting research where there is a very strongly supported hypothesis such that investigators are rather confident that they will find the true signal that they hypothesize. The only part of the calculations that needs to be adjusted to address such a situation is the strength of the *prior* on null hypothesis being true (*i.e.*, *f*(π) below).

It is well established that conclusions about the validity of a given hypothesis cannot be evaluated via hypothesis testing methods. This is because evaluation of whether to reject or accept a hypothesis, given the study data, does not answer the question that investigator should be intrinsically interested in. Rather than knowing whether the data is consistent or not consistent with a hypothesis at some arbitrarily defined probability, most investigators would rather know whether a hypothesis is true or understand how certain they can be that it is true. To answer such a question, one must adopt a Bayesian approach. Typically, the null hypothesis (of no association) is rejected with probability of α = 0.05. This does not mean that there is 95% chance that the alternative hypothesis is correct, nor does this imply that there is at most a 5% chance that it was not correct to reject the null hypothesis (*i.e*., there really is no association despite statistical significance, *viz. a viz.* false positive). The calculation that is helpful to address this relevant question for drawing quantitative conclusions involves the concept of False Positive Rate (FPR), estimated as FPR = α × π/(α × π + (1 − β) × (1 − π)), where α is the probability of type I error, β is the probability of type II error, and π is the probability that null hypothesis is true, *i.e.*, *prior* belief that there is no association.

We propose that it is also insufficient to plan whether studies should be conducted based only on rates of type I and II errors as is the custom. The probabilities as well as the consequences of false positive and negative results also need to be understood before one can judge whether a study is justified on statistical and ethical grounds. Our work is conceptually related to analysis by Phillips [4] on whether a given future study can produce a policy-meaningful change in estimate of an association but addresses different questions of false positives and negatives; the two arguments can be linked through consideration of impact of measurement error and exposure misclassification on effect estimates.

We will focus on what is commonly known as hypothesis-generating studies but note that our approach is applicable to all investigations that rely on statistical hypothesis tests and examination of confidence intervals. We shall incorporate into our analysis considerations of imperfection in exposure assessment that is virtually universal in observational research, such as epidemiology. For illustrative purposes, we consider specific examples of unmatched case-control studies with binary exposure classifiers, though the concepts apply more broadly, because (a) we work with logistic response (disease) model without any explicit consideration of retrospective case-control sampling and (b) non-differential exposure misclassification and measurement error have largely the same effect across many response models.

## 2. Methods and Results

### 2.1. What is a Hypothesis-Generating Study?

We defined hypothesis-generating study as a “large” investigation in the sense that it is powered to detect elusive effects with multiple exposures or outcomes assessed (e.g., a case-control study with 1500 case-control sets and several exposures or cases and controls from multiple outcomes evaluated against a single exposure). The investigators believe that only a few of the examined associations are true because for most exposure-outcome associations that are to be investigated they have a weak hypothesis (e.g., not supported by either previous empirical work or biologic considerations) or no particular reason to believe that studied exposures and outcomes are associated (e.g., Genome-Wide or Exposure-Wide Association Studies, such as those that correlate all exposures measured in NHANES with a single health outcome [5]). We focus on true associations that can be viewed as “weak”, e.g., with odds ratios of ≤2 for a typical categorization of exposure [6]. Throughout our presentation, we denote probability density function of random variable *z* as *f*(*z*).

The essential part of the calculation of the (posterior) distribution of the *False Positive Rate* (FPR) is Bayesian and involves stating the probability that H_0_ is true, *i.e.*, π, a *priori*. This allows us to quantify how unlikely a given exposure is to be truly associated with the outcome. Since pre-existing knowledge about the association is nearly always imperfect, we will consider a distribution of *f*(π) that is intended to capture a typical representation of hypothesis-generating studies, such that *f*(π)~Uniform(0.7, 1): “nearly all of them wrong” [1]. We also perform sensitivity analyses that either widen this distribution, which may be applicable to accidental releases of a known toxic chemical in which (a) hazards are largely unknown but biologically plausible or (b) narrow the distribution at the upper end of implausible as may be with a highly speculative association with no known mechanism. From the perspective of planning a study, instead of estimating FPR after data was observed, we can fix expected or desired effect size as a constant (denoted here as γ), since the calculation is conditioned on its specific value; however, in a specific application, investigators may wish to estimate FPR under uncertainty about γ by specifying *prior* distribution *f*(γ|π = 0). Of course, the *False Negative Rate* (FNR) may also be of inherent interest at the study planning stage; we revisit this issue later.

For illustrative purposes, we consider a simple study with one binary exposure *X* imperfectly classified as *W* and binary outcome *Y* that measured perfectly, so that the data can be analyzed by estimating odds ratios from a 2 × 2 table. The value α will be fixed at the traditional 0.05 and power is estimated for each desired effect size. We will also consider, without loss of generality, exposure that has prevalence of 0.3 (= p(X = 1)) (alternative exposure prevalence values generated similar conclusions in the main simulations).

### 2.2. True and Apparent Effect Size

Let γ (e.g., odds ratio (OR) in a case-control study) denote the smallest effect size greater than 1 that we plan to detect if the null hypothesis (H_0_: γ = 1) is false. The value of γ will depend on what we believe the true effect to be as well as how we think this effect will likely appear in a given study, e.g., as γ^*^ distorted from true value of γ by misclassification of binary exposure. It is always possible to postulate a plausible anticipated effect size before any data is collected or analyzed [7]. The specific case that we will consider is that of non-differential exposure misclassification, *viz.* *E*(|γ^*^|) < *E*(|γ|), when sensitivity (SN) and specificity (SP) of exposure classifier are such that 1 < SN + SP < 2.

### 2.3. When Exposure is Perfectly Classified

First, let us review what happens to FPR when exposure classification is perfect, so that *W* = *X* given that SN = SP = 1. In this case, barring any systematic biases, *E*(γ^*^) = *E*(γ), and we can proceed with calculation of FPR, fixing α = 0.05:

p((γ = 1|reject H_0_)|γ) = 0.05 × π/(0.05 × π + (1 − β) × (1 − π)), where *f*(π)~Uniform(0.7, 1)

**Figure 1:**
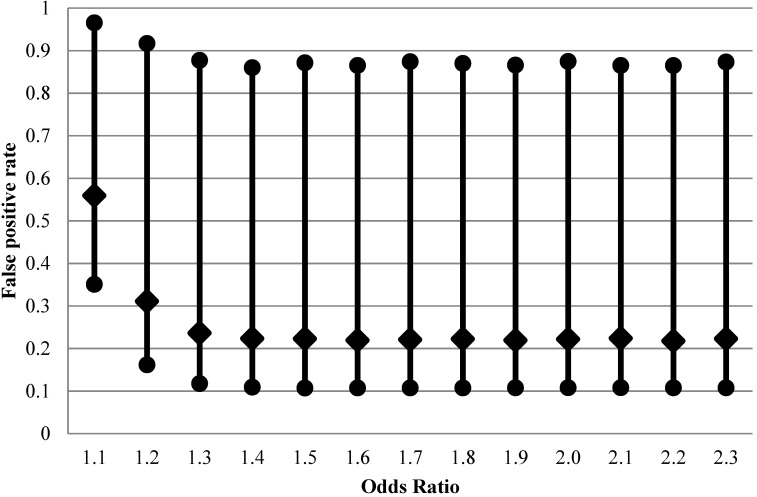
Impact of effect size on false positive rate (2.5th, 50th and 97.5th percentiles of 20,000 Monte-Carlo simulations) for fixed distribution of belief about probability of the effect being null (1500 case-control sets, type I error 5%, prevalence of exposure 30%, perfect exposure classification).

We conducted these calculations using expressions in Supplementary File 1 to derive β (*i.e.*, to estimate power for a fixed minimal detectable effect and sample size and for type I error) over a specified distribution of π in 20,000 Monte-Carlo simulations (implemented in R [8], see Supplementary File 2). The results of these calculations are presented in Figure 1 for a range of odds ratios exemplifying true weak associations starting from odds ratio of 1.1. There is a wide variation of FPR that can be occurring in each individual analysis, with 95% range between 10 and 90%. While we structure the main narrative of this article around simulation results, analytical expressions for distributions of FPR and FNR exist (Supplementary File 3) and are consistent with our results. Particularly, from the distributions of FPR and FNR and assuming *f*(π)~Uniform $\left(u_{1},\;u_{2}\right)$, we derive the analytical expressions of the expectation of FPR and FNR (*E*(FPR), *E*(FNR)), and the τ-th conditional quantile of FPR ($Q_{\tau }\left(FPR\right),Q_{\tau }\left(FNR\right))$:

$$E\left(FPR\right)=\frac{\alpha }{\alpha +\beta -1}-\frac{\alpha \left(1-\beta \right)}{\left(u_{2}-u_{1}\right){\left(\alpha +\beta -1\right)}^{2}}\text{log}\left(\frac{u_{2}\left(\alpha +\beta -1\right)+1-\beta }{u_{1}\left(\alpha +\beta -1\right)+1-\beta }\right)\;Q_{\tau }\left(FPR\right)=\frac{\left[\tau \left(u_{2}-u_{1}\right)+u_{1}\right]\alpha }{1-\beta -\left[\tau \left(u_{2}-u_{1}\right)+u_{1}\right]\left(1-\beta -\alpha \right)},\;0<\tau <1,\;E\left(FNR\right)=\frac{\beta }{\alpha +\beta -1}+\frac{\beta \left(1-\alpha \right)}{\left(u_{2}-u_{1}\right){\left(\alpha +\beta -1\right)}^{2}}\text{log}\left(\frac{u_{2}\left(1-\alpha -\beta \right)+\beta }{u_{1}\left(1-\alpha -\beta \right)+\beta }\right)\;Q_{\tau }\left(FNR\right)=\frac{\beta \left\{1-\left[u_{2}-\tau \left(u_{2}-u_{1}\right)\right]\right\}}{\beta +\left[u_{2}-\tau \left(u_{2}-u_{1}\right)\right]\left(1-\alpha -\beta \right)},\;0<\tau <1$$

These analytical expressions can be useful to study the trend of FPR and FNR when a certain parameter changes while the other parameters are fixed. We will look at it further in Section 2.4. The R code in Supplementary 3 can be used to perform theoretical percentile calculations of FPR and FNR.

### 2.4. Under the Misclassification of Binary Exposure

It is known that non-differential exposure misclassification can, on average, adversely affect power but is not expected to alter type I error rate (at least not in simple case we are considering). It is not realistic to expect perfect exposure classification in most observational studies, and this is a particularly germane expectation for hypothesis-generating studies. Perfect exposure measurement is can be exceedingly costly and, is usually unattainable anyway. To investigate the impact of non-differential misclassification of exposure on FPR, using fixed values of SN = 0.5 and SP = 0.9, which are realistic in many areas of epidemiology and certainly resemble the performance of exposure classifiers typically employed in occupational epidemiology [9,10,11]. We will explore a range of other values of SN and SP through theoretical calculations in sensitivity analyses.

In this context, estimation of FPR proceeds in two steps. First, we determine the expected value of effect size γ^*^ given its presumed true value γ and the extent of exposure misclassification (defined by SN and SP). Second, we use the derived γ^*^ value to calculate actual rate of type II error (β^*^) that is expected to be diminished due to exposure misclassification compared to the situation where exposure is perfectly classified. With these values in hand, we proceed to estimate FPR, fixing α = 0.05:

p((γ=1|reject H_0_)|γ^*^)=0.05×π/(0.05×π+(1-β^*^)×(1-π)), where *f*(π)~Uniform(0.7, 1)

**Figure 2:**
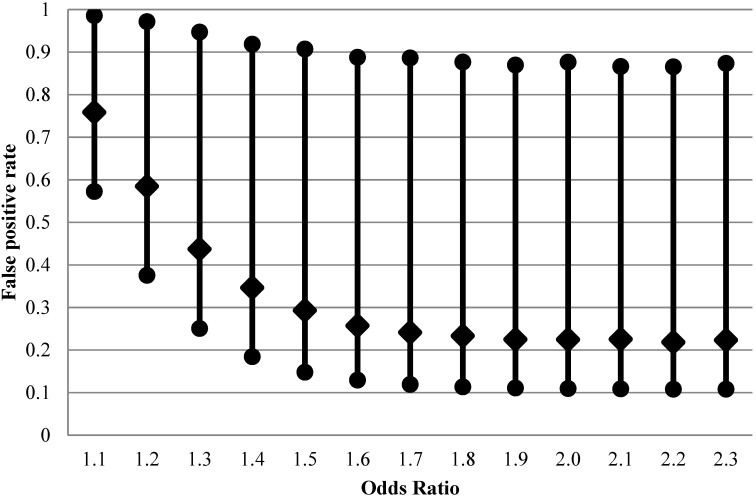
False positive rate (2.5th, 50th and 97.5th percentiles of 20,000 Monte-Carlo simulations) when exposure is imperfectly classified (sensitivity 0.5 and specificity 0.9) for fixed distribution of belief about probability of the effect being null (1500 case-control sets, type I error 5%, prevalence of exposure 30%, true rather than apparent odds ratios are shown).

The rest of the calculation is the same as in the situation with no exposure misclassification; mathematical details and implementation in the R environment are in Supplementary File 2. As can be seen from Figure 2, FPR is considerably higher than with perfect exposure assessment at design OR < 1.5, with expected values on the order of 30%–80% for the weaker effects.

When the nominal power of 80% is achieved at true OR = 1.6, the median of FPR is still at 25%, higher than the median when there is no exposure misclassification (21%, Figure 1). The apparent OR at this point is expected to be 1.3. *Thus, we can see that although non-differential exposure misclassification is expected to attenuate OR, the net effect is not only the well-known loss of ability to detect true effects (the false negative conclusion) but also a concurrent increase in false positives, due to inflation in the variability of the estimated effects.*

One important caveat to these arguments is that we consider exposures, corresponding to multiple exposure-outcome hypotheses, which are independent. This is rarely true in practice and affects FPR (e.g., [12]). The complex pattern of impact of measurement error on correlated covariates, with direction and magnitude of biases, are difficult to anticipate based on intuition alone (e.g., [13]).

**Figure 3:**
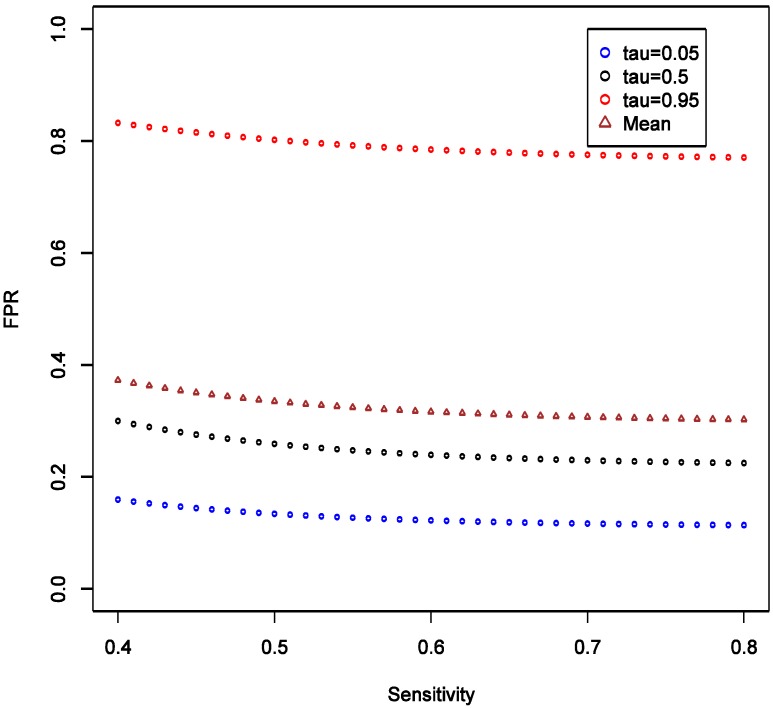
Theoretical calculations of the impact of varying sensitivity of exposure classifier while keeping specificity fixed at 0.9; tau (τ) = expected quintiles of the distribution for false positive rate (FPR); see text for details of other parameter settings.

With the analytic expressions related to the distributions of FPR and FNR in section 2.3, we can also study how parameters other than the values of target odds ratio may affect FPR and FNR. The R code required to generate these results is given as Supplementary File 4. To investigate the effect of SN, we consider the scenario with fixed OR at 1.6, and varying SN from 0.4 to 0.8, while keeping SP fixed at 0.9 and all other parameters as in the main example. We observed that the expectation, 5% and 95% quantiles of FPR are all quite robust to the changes in SN, and the variation of the distribution seems stable (Figure 3).

**Figure 4:**
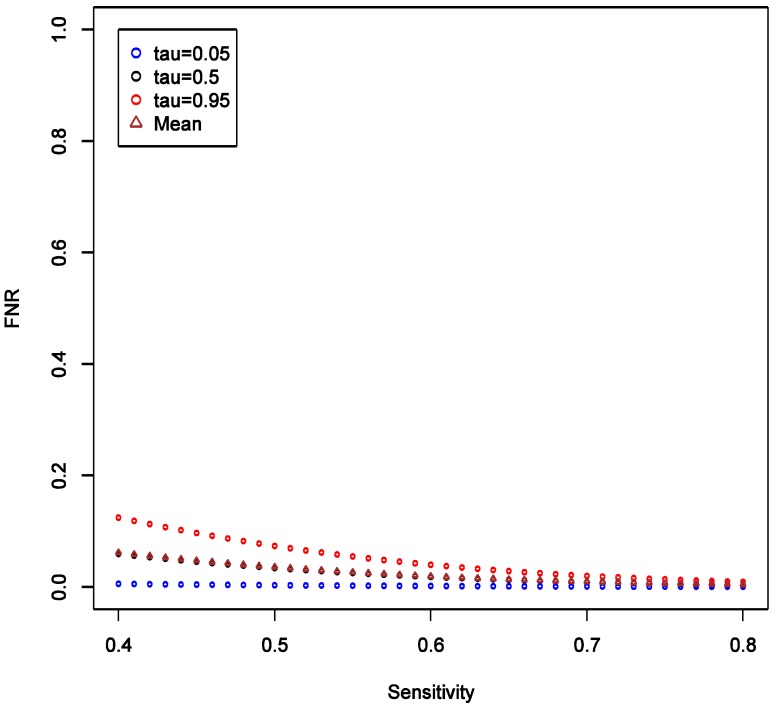
Theoretical calculations of the impact of varying sensitivity of exposure classifier while keeping specificity fixed at 0.9; tau (τ) = expected quintiles of the distribution for false positive negative (FNR); see text for details of other parameter settings.

This implies that the chance of false positive is insensitive to increase in SN of exposure classifier. With increasing SN, the FNR tends to decrease, and such decrease is more obvious in the higher quantiles (Figure 4), implying that our confidence in negative (null) results ought to grow as well. Unlike the effect on FPR, the variation of the distribution of FNR decreases substantially with increasing SN and FNR becomes considerably smaller than FPR (40% *vs.* <5%). Overall, we conclude that under such settings the confidence in null result ought to be greater than the confidence in a non-null result. This also confirms intuition that boosting sensitivity, so long as SP is maintained at a high value, has negligible impact on the chance of picking up true signal from noise case-control studies of uncommon exposures. As in the main example explored though simulations, FNR is calculated to be smaller than FPR across the settings.

**Figure 5:**
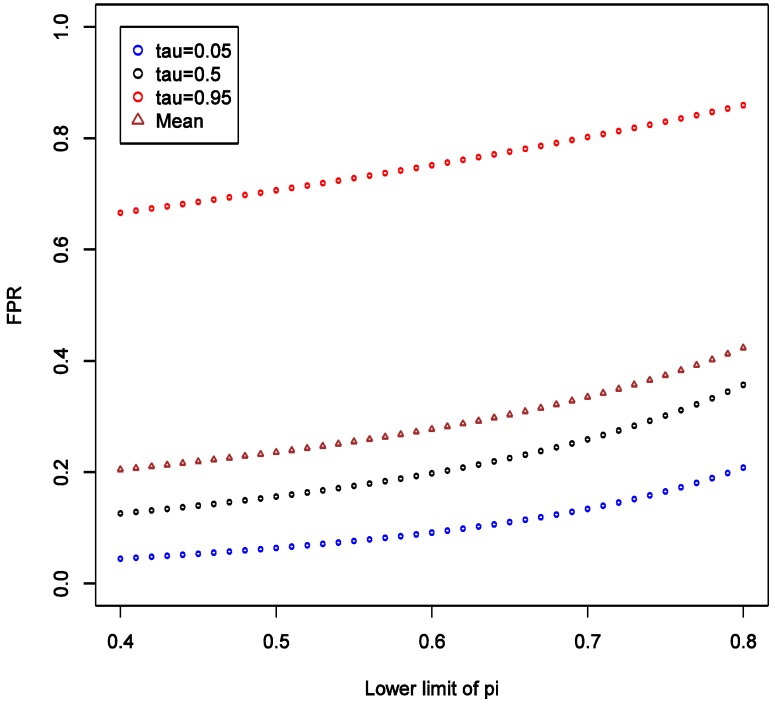
Theoretical calculations of the impact of varying strength belief in null hypothesis being true = pi (π); tau (τ) = expected quintiles of the distribution for false positive rate (FPR); see text for details of other parameter settings.

We also consider varying the lower limit of the prior distribution of π from 0.4 to 0.8 with the upper limit fixed at 1, while keeping all other parameters fixed as in the main example. This reflects varying degree of uncertainty about strength of hypothesis. We observed that as the lower limit increases, the mean, the 5% and 95% quantiles of FPR all increase, and the variation of the distribution appears to be stable (Figure 5).

**Figure 6:**
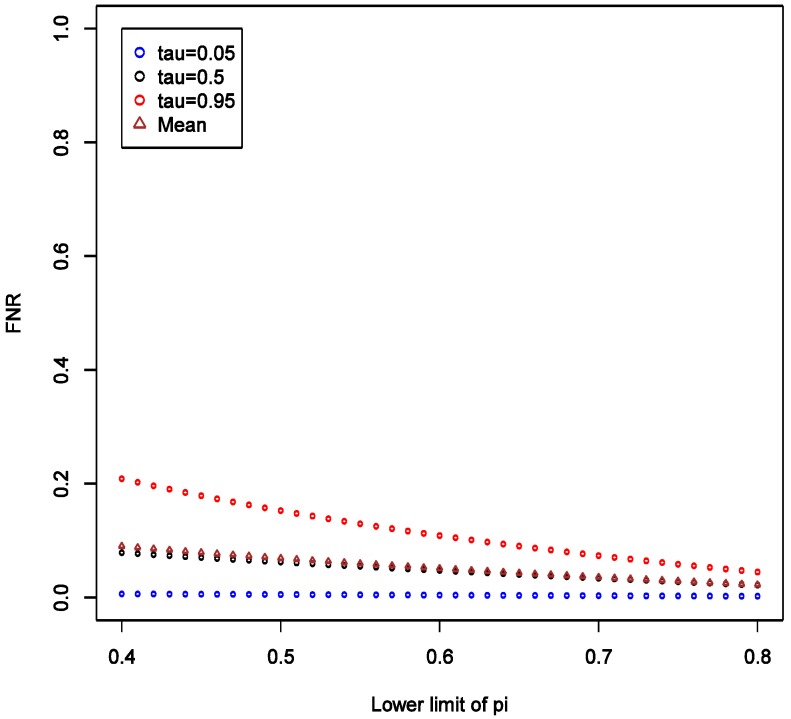
Theoretical calculations of the impact of varying strength belief in null hypothesis being true = pi (π); tau (τ) = expected quintiles of the distribution for false negative rate (FNR); see text for details of other parameter settings.

There is also a natural increase in certainty that a “statistically significant” result is a false positive as the prior on it being so becomes narrower with higher lower bound. The increasing lower limit of π distribution instead leads to a decrease in FNR especially at high quantiles, and the variation of distribution decreases (Figure 6). Again, we see by comparing Figure 5 and Figure 6 that FNR is smaller across wide range of settings than FPR—*i.e.*, more confidence can be placed on findings consistent with the null. The other conclusion that we can draw from these calculations is the confirmation that the stronger the prior (here in a sense that the distribution of π is narrow or has a higher lower limit), the less informative any data becomes in arriving at novel conclusions. Thus, it is very important to articulate such priors at least qualitatively, if not quantitatively in the motivation of research.

**Figure 7:**
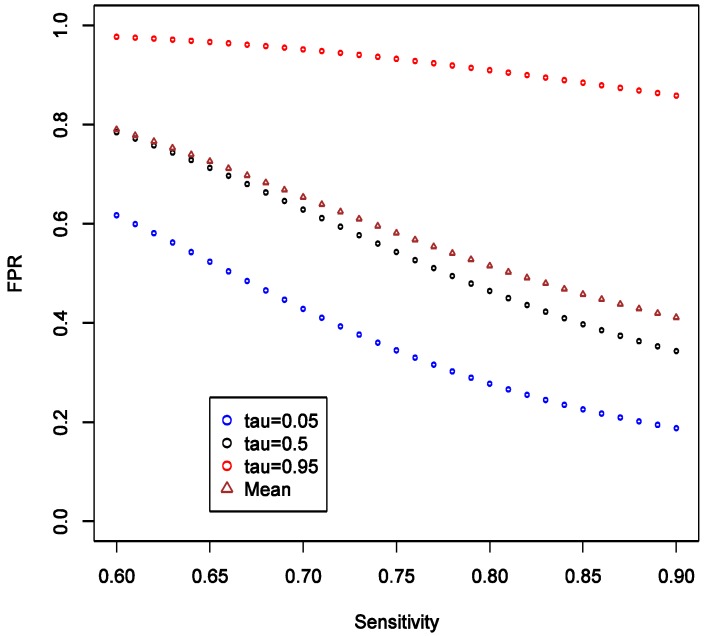
Theoretical calculations of the impact of varying sensitivity of exposure classifier while keeping specificity fixed at 0.5; tau (τ) = expected quintiles of the distribution for false positive rate (FPR); see text for details of other parameter settings.

**Figure 8:**
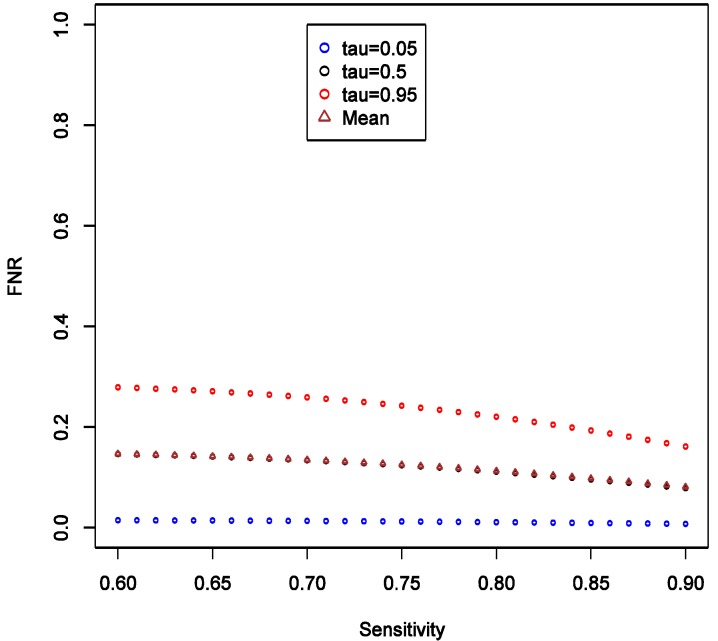
Theoretical calculations of the impact of varying sensitivity of exposure classifier while keeping specificity fixed at 0.5; tau (τ) = expected quintiles of the distribution for false negative rate (FNR); see text for details of other parameter settings.

Lastly, we investigated expected effect on FPR and FNR when SN is greater than SP, as might be expected when not all sources of a ubiquitous compound (e.g., polycyclic aromatic hydrocarbons, benzene, bisphenol-A, perfluorinated acids, *etc*.) are accounted for in exposure assessment. To accomplish this, we fixed SP = 0.5 and varied SN from 0.6 to 0.9, keeping all other parameters fixed as in the main example; see Supplementary File 5 for implementation details. Comparing Figure 3 and Figure 7, we note that having SP exceed sensitivity (at least in this setting) stabilizes FPR on average at around 0.4, while when SP is lower than SN, the average FPR tends to be large and only approaches 0.4 when SN is at the upper end of 0.9. As SN increases relative to SP the rates of both false positives (Figure 7) and false negatives (Figure 8) decline: the higher the quality of data the more reliable the conclusions. Yet again, FPR is higher than FNR, reflecting, most likely, influence of prior belief consistent with the exploratory nature of the study design we are considering. The distribution of FNR appears to be relatively insensitive to increase in SN relative to SP (Figure 8). Comparing Figure 4 and Figure 8, we also observe that FNR is more favorable when SP exceeds SN. This reinforces previous work that in case-control studies, SP should be kept higher than SN [14], though that conclusion was reached without consideration of FPR. *While our findings appear to be robust, we cannot be certain that exceptions to patterns we have reported do not occur. It is for this reason that we share with the reader all the tools necessary to evaluate their specific circumstances though the supplemental materials.*

### 2.5. How do These Calculations Help Make Decisions & Plan Studies?

Consider a case-control study with 1500 cases and 1500 controls, aiming to detect OR of at least 1.3, while allowing for 5% type I error rate and assuming prevalence of exposure of 30%. The conventional power calculation indicates that power is ~90% so we may feel encouraged, on statistical grounds, to pursue the study as one that can yield valuable insights into the hypothesis. However, after considering typical values of exposure misclassification assuming SN = 0.5 and SP = 0.9, the re-calculated power is only 36%! This is a more realistic assessment of whether to pursue such a weak effect as OR = 1.3.

Next, consider that among exposures that the study can evaluate with respect to the outcome, there is one that also has prevalence of 30% and with expected effect, based on prior literature, of at least OR of 1.6. Re-estimation of power under exposure misclassification is rather encouraging: 80%. But is this enough to proceed with the study? Let us further consider that prior evidence for this particular exposure causing the disease is “weak” as we posit in this paper (*i.e.*, *f*(π)~Uniform(0.7, 1)). Then our calculations reveal that we can expect the chance of false positive results for this association to be between 26 and 89%, with 95% probability. This is a far cry from the reassuring 5% figure implied (incorrectly!) by type I error rate. On the other hand, if we could be certain that *f*(π)~Uniform(0, 0.3), *i.e.*, there is at most 30% chance that null is true, then the 97.5th percentile of FPR can be estimated to be 2.5%. Clearly one should weigh carefully whether study is worth pursuing when one cannot be reasonably certain that “positive” findings are sufficiently trustworthy.

The terminology “reasonably certain” and “sufficiently trustworthy” was left intentionally vague at the end of previous paragraph. These quantities can only be appraised if we consider *the consequences* of drawing false conclusions (*i.e.*, acting on wrong assumption about how the world actually works). In the context of environmental and occupational epidemiology, this means assessing societal costs of making wrong decision. One can argue that decisions are never made based on a results of a single study but rather involve synthesis of a vast body of knowledge that may involve meta-analyses or pooled re-analyses of multiple investigations. We do not disagree with this general statement. However, it is always worth considering whether one more study will change how we view the world (e.g., accept or reject conjecture) or act on it (e.g., conduct more research, take a product off the market, impose regulations on exposure or a product). Even if a study does not lead to any material change, the lack of change is in fact consequence of a study. Thus, every investigation that is informative (*i.e.*, not of such poor quality that it contains no information) has consequences. Once we accept this, we shall be closer to conducting useful and responsible research: every well-conducted study counts and the question only is just how much it counts or matters. *We shall not develop this argument further in the current manuscript but challenge ourselves and the reader to reflect on these matters and attempt to address them in specific applied problems that we face in epidemiology and public health.*

**Figure 9:**
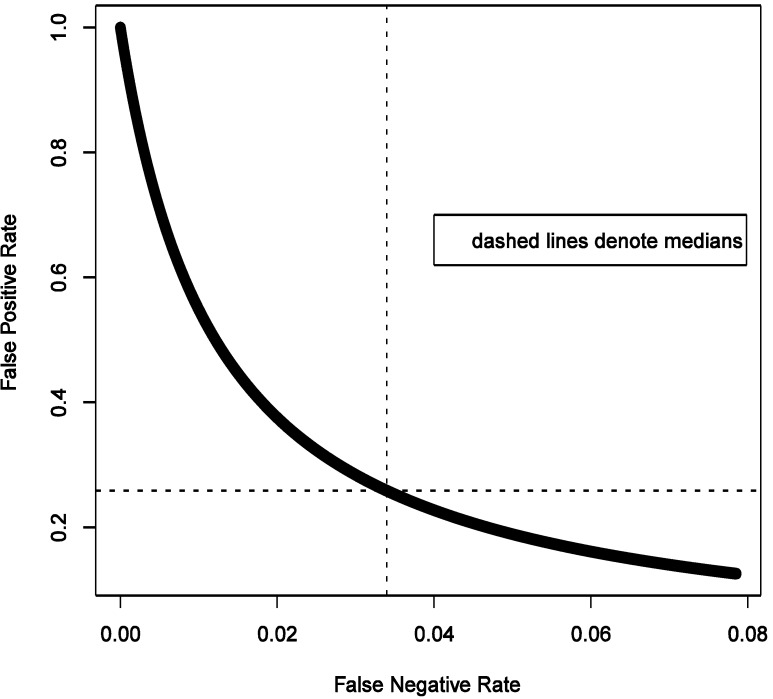
Illustrated relationship between false positive and false negative rates in a hypothesis generating study: 20,000 Monte-Carlo simulations when exposure is imperfectly classified (sensitivity 0.5 and specificity 0.9) for fixed distribution of belief about probability of the effect being null (1500 case-control sets, type I error 5%, prevalence of exposure 30%, true odds ratio 1.6).

Furthermore, if there is a cause of a specific outcome among studied exposures, then it is also important to consider the *consequences of a false negative finding*. We start with belief that at least some of the exposures we study do cause the outcome. To formally consider this, we need to calculate false negative rate estimated as FNR = β × (1 − π)/(β × (1 − π) + (1 − α) × π) under the same conditions as calculation of FPR. For a hypothesis generating study that we describe above in the simulations that anchor the main narrative, with OR = 1.6 and imperfect exposure assessment, FNR can be estimated to have median of 3.4% with 95% of the values falling between 0.2 and 7.6% (procedure for estimation is analogous to FPR and is summarized in code in Supplementary File 2). Figure 9 shows that FNR is relatively invariant and small compared to FPR in this situation. This confirms intuition that false negative findings are unlikely in a hypothesis-generating study and such work can be more reassuring than not when it fails to uncover associations. This observation is consistent with theoretical expectations derived in section 2.4 and presented in Figure 3, Figure 4, Figure 5 and Figure 6.

Let us explore some implications numerically in hypothetical study that has been the focus of this article. For this purpose, we fix OR = 1.6, α = 0.05, assumed exposure misclassification as defined above. The expected probabilities of the four study outcomes are summarized in the Table 1 (from 20,000 MC simulations).

It is curious to note that as FPR exceeds FNR (Figure 9), we can also expect on average more false positive results (4%) than false negative ones (3%); the certainty of false positive result is greater as seen from much narrower variability of the estimate (±1% *vs.* ±3%). True negative findings are expected to dominate results of study we are considering. This is consistent with the notion that the likelihood of there being a true association is small in a hypothesis-generating study.

**Table 1:** Expected rates of different outcomes of study considered in Figure 9 (percent: median, (2.5 to 97.5%)).

The truth	*Acceptance of H_0_*	
	Reject	Do not Reject
*H_0_ is* true	FP	TN
	**4**	**80**
	(3–5)	(67–94)
*H_0_ is* false	TP	FN
	**12**	**3**
	(1–24)	(0.1–6)

We argue that these issues must be addressed while applying for funding. *i.e.*, motivating research on a given topic. Power calculations can be corrected for expected or suspected misclassification in a given setting. The question of just how good the exposure estimates are is usually debated and presented in any grant proposal, with the applicants maintaining a fine balance between claiming that exposure assessment methods are useful without overstating their utility. At the very least, the applicants must defend a position that exposure assessment is adequate and in doing so they can refer to previous research with similar exposure assessment tools—there is plenty of relevant literature most common applications such as assessment of exposure to in case-control studies (e.g., see [15]). It is typically possible to derive sensible guesses on SN and SP from the existing research, even if one has to ask for experts to guess such values, e.g., see Liu *et al* [10]. If the information on quality of exposure assessment is completely lacking or yields calculations that are too uncertain to be useful, this argues for a grant to be submitted to develop and test exposure assessment tool. Such projects, sadly, are difficult to find support for but it has been argued by many that they are absolutely essential to progress in the field. Perhaps our work will add to substantial body of evidence that argues for better understating of exposure assessment methods and their improvement (impossible without assessments of reliability and validity).

It is also important to reflect what our calculations mean for interpretation of studies. It is possible to conduct Bayesian analysis of “hypothesis-generating” studies that accounts for the likelihood of any specific association being true (*i.e.*, the distribution of π) although developing such a method is outside of scope of current work. Outside of application of formal methods for determining false discovery rate, *etc.*, it is also possible to appraise such results qualitatively in light of the expected false positives and negatives (as in Table 1), as one does with the simplest corrections for multiple comparisons in hypothesis testing (*i.e.*, expecting 1/20 associations be by due to chance with statistical significance is set at 5%). It is reasonable to argue that our analytical framework more naturally applies to “confirmatory” rather than “exploratory” studies if one accepts that all possible hypotheses already exist [1]. From this perspective, we would be interested in evaluating both the strength of exposure-outcome association (θ, typically a coefficients of regression used to estimate the disease model) supported by the data and consistent with hypothesized effect, and the probability that the null effect is true given some observed association in the data (*i.e.*, π). Formally, we would be interested in the *posterior* distributions *f*(θ|data) and *f*(π|data). Given that, intuitively, the knowledge about value of θ is related to knowledge of how likely it is equal exactly to the null value, the *posterior* distribution of interest, *f*(θ,π|data), is anticipated to be a mixture distribution with point mass density at null governed by *f*(π|data), such that, say δ~*Bernoulli*(π) is an indicator variable of whether θ is null and a constant when δ = 1, or θ is non-null and follows a continuous distribution, such as normal for log-OR, when δ = 0. In this case we need to view π as a hyper-parameter in a Bayesian hierarchical model. By Bayes theorem, the model that we need to then consider is *f*(θ,π|data) = *f*(data| θ,π) *f*(θ) *f*(π)/*f*(data), where *f*(data|θ,π) is the likelihood, and *f*(θ) is the *prior* on the disease model parameter(s). The analytic approach should be able incorporate correction for measurement error and risk factor misclassification [13], as well as latent confounding, making it a perhaps a very useful general framework for a wide range of data analysis problems common in epidemiology. We will not develop this argument further at present.

## 3. Conclusions

We have demonstrated that in planning a study it is not sufficient to consider type I and type II error rates but it is imperative to understand the quality of data that can be gathered as well as strength of the hypothesis. To do so, one must confront the problem of false positive and false negative findings as well as “quality of information” (e.g., exposure misclassification). We illustrate specifically the problems with under-estimation of false positive rates when imperfect exposure metrics are treated as if they are free of errors, as is usually the case. In doing so, we created a set of tools that should enable future investigators to calculate whether specific a hypothesis (already generated [1]) are either worth pursuing or what study size and design are needed to obtain an informative answer to a qualitative question about truth of the hypothesis. Such a question should consider the costs of alternative outcomes of a particular investigation, the matter that we left for future exploration. We suggest that instead of investing energy into understanding credibility of dubious hypotheses, applied disciplines such as epidemiology should instead focus their attention on understanding consequences of pursuing specific hypotheses as well, as evaluating the magnitude of effect estimates, so long as it is considered to what precise degree an effect estimate that suggests an association may yet prove to be a false signal.

## Supplementary Files

Supplementary File 1
