# Supplementary material for: Effects of Non-Differential Exposure Misclassification on False Conclusions in Hypothesis-Generating Studies
Source: Int J Environ Res Public Health. 2014 Oct 21;11(10):10951–66. doi: 10.3390/ijerph111010951 (PMC4211016; doi:10.3390/ijerph111010951)
Supplement: Supplementary File 1 [file ijerph-11-10951-s001.pdf]

Supplementary Information

Effects of Non-Differential Exposure Misclassification on False Conclusions in Hypothesis-Generating Studies

---

Supplementary File 1: Power of Test of Log-Odds Ratio in Presence of Exposure Misclassification

Non-Differential Misclassification of Binary Exposure

Note that w is observed and x is true, and the outcome variable y is binary. We consider binary w and x. Assume

$$P(y=1|x) = \exp(\beta_0+\beta_1 x)\,/(1+\exp(\beta_0+\beta_1 x))$$

$$P(y=1|w) = \exp(b_0+b_1 w)\,/(1+\exp(b_0+b_1 w))$$

$$P(y|x,w) = P(y|x)$$

We have

$$P(y=1|w) = P(y=1,w)/P(w)$$

in which

$$P(y=1,w) = P(y=1,w,x=0) + P(y=1,w,x=1)$$

$$= P(y=1|x=0)\times P(w|x=0)\times P(x=0)$$

$$+ P(y=1|x=1)\times P(w|x=1)\times P(x=1)$$

$$P(w) = P(w|x=0)p(x=0) + p(w|x=1)p(x=1)$$

Given β_0, β_1, we have the following equations to solve b_0, b_1.

$$\frac{\exp(b_0)}{1+\exp(b0)} = \frac{\frac{\exp(\beta_0)}{1+\exp(\beta_0)}P(w=0|x=0)P(x=0) + \frac{\exp(\beta_0+\beta_1)}{1+\exp(\beta_0+\beta_1)}P(w=0|x=1)P(x=1)}{P(w=0|x=0)P(x=0) + P(w=0|x=1)P(x=1)}$$

$$\frac{\exp(b_0+b1)}{1+\exp(b0+b1)} = \frac{\frac{\exp(\beta_0)}{1+\exp(\beta_0)}P(w=1|x=0)P(x=0) + \frac{\exp(\beta_0+\beta_1)}{1+\exp(\beta_0+\beta_1)}P(w=1|x=1)P(x=1)}{P(w=1|x=0)P(x=0) + P(w=1|x=1)P(x=1)}$$

Because $= P(w=1|x=1), SP = P(w=0|x=0)$, it leads to

$$\frac{\exp(b_0)}{1+\exp(b0)} = \frac{\frac{\exp(\beta_0)}{1+\exp(\beta_0)}SP\times P(x=0) + \frac{\exp(\beta_0+\beta_1)}{1+\exp(\beta_0+\beta_1)}(1-SN)\times P(x=1)}{SP\times P(x=0) + (1-SN)\times P(x=1)}$$

$$\frac{\exp(b_0+b1)}{1+\exp(b0+b1)} = \frac{\frac{\exp(\beta_0)}{1+\exp(\beta_0)}(1-SP)\times P(x=0)+\frac{\exp(\beta_0+\beta_1)}{1+\exp(\beta_0+\beta_1)}SN\times P(x=1)}{(1-SP)\times P(x=0)+SN\times P(x=1)}$$

Solving the above equations we have

$$b_0=\log\{\frac{\frac{\exp(\beta_0)}{1+\exp(\beta_0)}SP\times P(x=0)+\frac{\exp(\beta_0+\beta_1)}{1+\exp(\beta_0+\beta_1)}(1-SN)\times P(x=1)}{\frac{SP\times P(x=0)}{1+\exp(\beta_0)}+\frac{(1-SN)\times P(x=1)}{1+\exp(\beta_0+\beta_1)}}\}$$

$$b_1=\log\{\frac{\frac{\exp(\beta_0)}{1+\exp(\beta_0)}(1-SP)\times P(x=0)+\frac{\exp(\beta_0+\beta_1)}{1+\exp(\beta_0+\beta_1)}SN\times P(x=1)}{\frac{(1-SP)\times P(x=0)}{1+\exp(\beta_0)}+\frac{SN\times P(x=1)}{1+\exp(\beta_0+\beta_1)}}\} - b_0$$

The hypothesis testing can be formulated as

$$H_0: \beta_1 = 0 \quad VS \quad H_1: \beta_1 \neq 0$$

As given in Demidenko (2007) (Sample size determination for logistic regression revisited),

$$Power = \Phi\left(-Z_{1-\frac{\alpha}{2}}+\sqrt{n}\frac{\beta_1}{\sqrt{V_{22}}}\right)+\Phi\left(-Z_{1-\frac{\alpha}{2}}-\sqrt{n}\frac{\beta_1}{\sqrt{V_{22}}}\right)$$

where $\beta=(\beta_0,\beta_1),\ V=I_\beta^{-1}$ is the asymptotic variance-covariance matrix of the $\sqrt{n}\hat{\beta}_{ML}$, I_β is the Fisher information matrix for β. More specifically, I_β has the following expression

$$I_\beta=\begin{pmatrix} I_{\beta_0} & I_{\beta_0\beta_1} \\ I_{\beta_0\beta_1} & I_{\beta_1}\end{pmatrix}$$

with $I_{\beta_0}=E_x\left\{\frac{\exp(\beta_0+\beta_1 x)}{(1+\exp(\beta_0+\beta_1 x))^2}\right\}$, $I_{\beta_0\beta_1}=E_x\left\{\frac{\exp(\beta_0+\beta_1 x)}{(1+\exp(\beta_0+\beta_1 x))^2}x\right\}$, $I_{\beta_1}=E_x\left\{\frac{\exp(\beta_0+\beta_1 x)}{(1+\exp(\beta_0+\beta_1 x))^2}x^2\right\}$.

For binary x, it is easy to calculate the I_β matrix with given values of β_0,β_1 in H_1. It is easy to see that $I_{\beta_0\beta_1}=I_{\beta_1}$.

Then we have

$$V=\begin{pmatrix}\frac{1}{I_{\beta_0}-I_{\beta_1}} & \frac{1}{I_{\beta_1}-I_{\beta_0}} \\ \frac{1}{I_{\beta_1}-I_{\beta_0}} & \frac{I_{\beta_0}}{I_{\beta_1}(I_{\beta_0}-I_{\beta_1})}\end{pmatrix}$$

Combined with the equation for power, we have

$$V_{22}=\frac{I_{\beta_0}}{I_{\beta_1}(I_{\beta_0}-I_{\beta_1})}=\frac{(1+\exp(\beta_0))^2}{\exp(\beta_0)\,P(x=0)}+\frac{(1+\exp(\beta_0+\beta_1))^2}{\exp(\beta_0+\beta_1)\,P(x=1)}$$

When we are doing hypothesis testing with measurement errors in x, we need to replace the β_0,β_1 in the above power expression by b_0,b_1.

Supplementary File 2: R-Environment Implementation of Calculations Used to Generate the Figures in the Article that Pertain to False Positive and False Negative Rates

```
#######################
########inputs##########
#######################
#parameters
set.seed(3.1416926)
n < −3000   #size of planned study 1500 cases and 1500 controls
SN < −0.5
SP < −0.9
px1 < −0.3
alpha < −0.05 #type I error
Z < −qnorm((1 − alpha/2), mean = 0, sd = 1, lower.tail = TRUE, log.p = FALSE)
beta0 < −0 #logit parameter of background rate of outcome/disease
beta1 < −log(1.6) #presumed true log-OR

################
#generate “data”#
################
k < −20000 # number of Monte Carlo samples
####parameters of prior on null being true f(pi)~Uniform(ipH0.a, ipH0.b)
ipH0.a < −0.7 #lower bound PROBABILITY null is true
ipH0.b < −1.0 #upper bound PROBABILITY null is true
pH0 < −runif(k, ipH0.a, ipH0.b)  #prior on pi

####################################
##########Monte-Carlo sampling##########
####################################

#########################################
#calculation of power WITHOUT measurement error
#######################################

V22_nome < −(1 + exp(beta0))^2/(exp(beta0) × (1 − px1)) + (1 + exp(beta0 + beta1))^2/(exp(beta0 + beta1) × px1)
q_nome1 < −(−Z + sqrt(n) × beta1/sqrt(V22_nome))
q_nome2 < −(−Z − sqrt(n) × beta1/sqrt(V22_nome))
power_nome < −pnorm(q_nome1) + pnorm(q_nome2)
mean(power_nome)

#false positive rate
FPR < −alpha × pH0/(alpha × pH0 + power_nome × (1 − pH0))
```

```
#false negative rate
FNR < −(1 − power_nome) × (1 − pH0)/((1 − power_nome) × (1 − pH0) + (1 − alpha) × (pH0))

#given beta solve for b (estimate of beta)
b0n < −SP × (1 − px1) × exp(beta0)/(1 + exp(beta0)) + (1 − SN) × px1 × exp(beta0 + beta1)/(1 +
exp(beta0 + beta1))
b0d < −SP × (1 − px1)/(1 + exp(beta0)) + (1 − SN) × px1/(1 + exp(beta0 + beta1))
b0 < −log(b0n/b0d)
b1n < −(1 − SP) × (1 − px1) × exp(beta0)/(1 + exp(beta0)) + SN × px1 × exp(beta0 + beta1)/(1 +
exp(beta0 + beta1))
b1d < −(1 − SP) × (1 − px1)/(1 + exp(beta0)) + SN × px1/(1 + exp(beta0 + beta1))
b1 < −log(b1n/b1d) − b0

###########################################
#calculation of power WITH measurement error
###########################################

V22 < −(1 + exp(b0))^2/(exp(b0) × (1 − px1)) + (1 + exp(b0 + b1))^2/(exp(b0 + b1) × px1)
q1 = −Z + sqrt(n) × b1/sqrt(V22)
q2 = −Z − sqrt(n) × b1/sqrt(V22)
power = pnorm(q1) + pnorm(q2)

#false positive rate
FPRpq < −alpha × pH0/(alpha × pH0 + power × (1 − pH0))

#false negative rate
FNRpq < −(1 − power) × (1 − pH0)/((1 − power) × (1 − pH0) + (1 − alpha) × (pH0))

###########################################end#########################################
###
```

Supplementary File 3: Distributions of False Positive Rates (FPR) and False Negative Rates (FNR)

For FPR, FPR = $\frac{\alpha\pi}{\alpha\pi+(1-\beta)(1-\pi)}$.

We have for $0 \leq t \leq 1$

$$P(FPR \leq t) = P\left(\frac{\alpha\pi}{\alpha\pi + (1-\beta)(1-\pi)} \leq t\right) = P(\pi \leq \frac{(1-\beta)t}{\alpha(1-t)+(1-\beta)t})$$

If $\pi \sim Unif(0.7, 1)$,

$$P(FPR \leq t) = \frac{\frac{(1-\beta)t}{\alpha(1-t)+(1-\beta)t} - 0.7}{0.3} =. \text{ (Distribution function of FPR)}$$

There is actually a lower bound of FPR: $FPR \geq \frac{0.7\alpha}{0.3(1-\beta)+0.7\alpha}$

Correspondingly, the τ-th quantile of FPR can be calculated through

$$\frac{(1-\beta)t}{\alpha(1-t)+(1-\beta)t}=0.3\tau+0.7$$

Then we have

$$Q_\tau(FPR)=\frac{(0.3\tau+0.7)\alpha}{(1-\beta)-(0.3\tau+0.7)(1-\beta-\alpha)}$$
$$=\frac{(0.3\tau+0.7)\alpha}{[1-(0.3\tau+0.7)](1-\beta)+(0.3\tau+0.7)\alpha}$$

- Lowering type I error α, helps lowering FPR for all τ.
- The influence from lowering α is more substantial when the power $1-\beta$ is greater.

If we look the mean of FPR

$$E(FPR)=\int_{0.7}^{1}\frac{\alpha\pi}{\alpha\pi+(1-\beta)(1-\pi)}\times\frac{1}{0.3}d\pi$$
$$=\frac{\alpha}{\alpha+\beta-1}-\frac{\alpha(1-\beta)}{0.3(\alpha+\beta-1)^2}\log(\frac{\alpha}{0.3(1-\beta)+0.7\alpha})$$

For FNR, $FNR=\frac{\beta(1-\pi)}{\beta(1-\pi)+(1-\alpha)\pi}$

We have for $0\le t\le 1$,

$$P(FNR\le t)=P\left(\frac{\beta(1-\pi)}{\beta(1-\pi)+(1-\alpha)\pi}\le t\right)=P(\pi\ge\frac{\beta(1-t)}{\beta(1-t)+(1-\alpha)t})$$

If $\pi\sim Unif(0.7,1)$,

$$P(FNR\le t)=\frac{(1-\alpha)t}{0.3[\beta(1-t)+(1-\alpha)t]}.\text{ (Distribution function of FNR).}$$

There is actually an upper bound of FNR: $FNR\le\frac{0.3\beta}{0.3\beta+0.7(1-\alpha)}$.

Correspondingly, the τ-th quantile of FNR can be calculated through

$$\frac{(1-\alpha)t}{0.3[\beta(1-t)+(1-\alpha)t]}=\tau$$

Then we have

$$Q_\tau(FNR)=\frac{0.3\tau\beta}{(1-\alpha)(1-0.3\tau)+0.3\tau\beta}$$

The influence of α is quite limited for FNR once β is substantially larger than α.

If we look the mean of FNR

$$E(FNR)=\int_{0.7}^{1}\frac{\beta(1-\pi)}{\beta(1-\pi)+(1-\alpha)\pi}\times\frac{1}{0.3}d\pi$$
$$=\frac{\beta}{\alpha+\beta-1}-\frac{\beta(1-\alpha)}{0.3(\alpha+\beta-1)^2}\log(\frac{0.7(1-\alpha)+0.3\beta}{1-\alpha})$$

Implementation of calculations in R

```
###########################################
##############FPR#######################
###########################################
###FPR calculation formular##
pfFPR = function(a, p, x){
a × x/(a × x + p × (1 − x))}

x = runif(100000, 0.7, 1)

###mean FPR calculation###
mvFPR = function(a, p){
a/(a − p) – p × a/(a − p )/(a − p) × log(a/(p + (a − p) × 0.7))/0.3}

###tau-th quantile FPR calculation##
qrFPR = function(a, p, tau){
(0.3 × tau + 0.7) × a/(p − (0.3 × tau + 0.7) × (p − a))}

###distribution function for FPR##
dFPR = function(a, p, x){
(p × x/(a × (1 − x) + p × x) − 0.7)/0.3}
a = 0.05
p = 0.6
dFPR1 = function(x){
dFPR(a, p, x)
}
curve(dFPR1, xlim = c(0, 1))

###########################################
##############FNR#######################
###########################################
###distribution function for FNR###
dFNR = function(a, p, x){
(1 − a)/0.3/((1 − p) × (1 − x) + (1 − a))
}
a = 0.05
p = 0.6
dFNR1 = function(x){
dFNR(a, p, x)
}

Upb = 0.3 × (1 − p)/(0.3 × (1 − p) + 0.7 × (1 − a))
curve(dFNR1, xlim = c(0, upb))
```

```
###FNR calculation formular##
pfFNR = function(a, p, x){
 (1 − p) ×(1 − x)/((1 − p) ×(1 − x) + (1 − a) ×x)}
###tau-th quantile FNR calculation##
qrFNR = function(a, p, tau){
 0.3 ×tau ×(1 − p)/((1 − a) ×(1 − 0.3 ×tau) + 0.3 ×tau ×(1 − p))}

###mean FNR calculation###
mvFNR = function(a, p){
 (1 − p)/(a − p) − (1 − p) ×(1 − a)/0.3/(a − p)/(a − p) ×log((0.7 ×(1 − a) + 0.3 ×(1 − p))/(1 − a))}

###########test##########
a = 0.05
p = 0.6
x = runif(100000, 0.7, 1)
v = pfFNR(a, p, x)
quantile(v, p = c(0.05, 0.25, 0.5, 0.75, 0.95))
qrFNR(a, p, c(0.05, 0.25, 0.5, 0.75, 0.95))
mean(v)
mvFNR(a, p)
```

Supplementary File 4: Theoretical Calculations of the Distributions of False Positive Rates (FPR) and False Negative Rates (FNR) while Varying Sensitivity (SN) and Specificity (SP) and Lover limit of Prior on π (pi)—Sensitivity Analyses

```
#POWER
###setting###
n < −3000   #size of planned study 1500 cases and 1500 controls
SN < −0.5 #sensitivity
SP < −0.9 #specificity
px1 < −0.3 #prevalence of exposure
alpha < −0.05 #critical p-value for hypothesis testing
##
beta0 < −0 #logit parameter of background rate of outcome/disease
beta1 < −log(1.6) #presumed true log-OR

####parameters of prior on null being true f(pi)~Uniform(ipH0.a, ipH0.b)
ipH0.a < −0.7 #lower bound PROBABILITY null is true
ipH0.b < −1.0 #upper bound PROBABILITY null is true

####Function to calculate the power with input beta0, beta1, SN, SP###
powercal = function(beta0, beta1, SN, SP){
```

```
  b0n < −SP × (1 − px1) × exp(beta0)/(1 + exp(beta0)) + (1 − SN) × px1 × exp(beta0 + beta1)/(1 +
exp(beta0 + beta1))
  b0d < −SP × (1 − px1)/(1 + exp(beta0)) + (1 − SN) × px1/(1 + exp(beta0 + beta1))
  b0 < −log(b0n/b0d)
  b1n < −(1 − SP) × (1 − px1) × exp(beta0)/(1 + exp(beta0)) + SN × px1 × exp(beta0 + beta1)/(1 +
exp(beta0 + beta1))
  b1d < −(1 − SP) × (1 − px1)/(1 + exp(beta0)) + SN × px1/(1 + exp(beta0 + beta1))
  b1 < −log(b1n/b1d) − b0

  V22 < −(1 + exp(b0))^2/(exp(b0) × (1 − px1)) + (1 + exp(b0 + b1))^2/(exp(b0 + b1) × px1)
  Z < −qnorm((1 − alpha/2), mean = 0, sd = 1, lower.tail = TRUE, log.p = FALSE)
  q1 = −Z + sqrt(n) × b1/sqrt(V22)
  q2 = −Z − sqrt(n) × b1/sqrt(V22)
  power = pnorm(q1) + pnorm(q2)

  return(power)
}

######################DISTRIBUTION OF FALSE POSITIVE RATE (FPR)

####funcion to calculate the tau-th quantile of FPR###
####power can be calculated by powercal function first###

FPRfun = function(tau, u1, u2, alpha, power){
 (tau × (u2 − u1) + u1) × alpha/(power − (tau × (u2 − u1) + u1) × (power − alpha))
}

##function to calculate the expectation of FPR###
FPRavg = function(u1, u2, alpha, power){
  alpha/(alpha − power) – alpha × power/(u2 − u1)/((alpha − power)^2) × log((u2 × (alpha − power) +
power)/(u1 × (alpha − power) + power))
}

##############FPR: FIGURE 3################################################
######varying SN 0.4 to 0.8 by 0.01####
SNv = seq(0.4, 0.8, by = 0.01)

powerv = rep(0, length(SNv))
for(i in 1:length(SNv)){
 powerv[i] = powercal(beta0, beta1, SNv[i], SP)
}

FPRv = matrix(0, ncol = 4, nrow = length(SNv))
taus = c(0.05, 0.5, 0.95)
```

```
for(i in 1:length(SNv)){
  for(j in 1:length(taus)){
    FPRv[i, j] = FPRfun(taus[j], ipH0.a, ipH0.b, alpha, powerv[i])
  }
  FPRv[i, j + 1] = FPRavg(ipH0.a, ipH0.b, alpha, powerv[i])
}

plot(SNv, FPRv[,2], cex = 0.5, xlab = “Sensitivity”, ylab = “FPR”, ylim = c(0,1))
points(SNv, FPRv[,1], cex = 0.5, col = “blue”)
points(SNv, FPRv[,3], cex = 0.5, col = “red”)
points(SNv, FPRv[,4], cex = 0.5, col = “brown”, pch = 2)
legend(0.7, 1, c(“tau = 0.05”, “tau = 0.5”, “tau = 0.95”, “Mean”), col = c(“blue”, “black”, “red”,
“brown”), pch = c(rep(1, 3), 2))

#############FPR: FIGURE 5############################################
###varying lower limit from 0.4 to 0.8####

u1v = seq(0.4, 0.8, by = 0.01)

power = powercal(beta0, beta1, SN = 0.5, SP)

FPRv = matrix(0, ncol = 4, nrow = length(u1v))
taus = c(0.05, 0.5, 0.95)

for(i in 1:length(u1v)){
  for(j in 1:length(taus)){
    FPRv[i, j] = FPRfun(taus[j], u1v[i], ipH0.b, alpha, power)
  }
  FPRv[i, j + 1] = FPRavg(u1v[i], ipH0.b, alpha, power)
}

plot(u1v, FPRv[,2], cex = 0.5, xlab = “Lower limit of pi”, ylab=“FPR”, ylim=c(0,1))
points(u1v, FPRv[,1], cex = 0.5, col = “blue”)
points(u1v, FPRv[,3], cex = 0.5, col = “red”)
points(u1v, FPRv[,4], cex = 0.5, col = “brown”, pch = 2)
legend(0.4, 1, c(“tau = 0.05”, “tau = 0.5”, “tau = 0.95”, “Mean”), col = c(“blue”, “black”, “red”,
“brown”), pch = c(rep(1, 3), 2))
#####################DISTRIBUTION OF NEGATIVE POSITIVE RATE (FNR)

#####Function to calculate the tau-th quantile of FNR######
FNRfun = function(tau, u1, u2, alpha, power){
  (1 − power) × (1 −(u2 − tau × (u2 − u1)))/(1 – power + (u2 − tau × (u2 − u1)) × (power − alpha))
}
```

```
###Function to calculate the expectation of FNR####
FNRavg = function(u1, u2, alpha, power){
(1 − power)/(alpha − power) + (1 − power) × (1 − alpha)/(u2 − u1)/((alpha − power)^2) × log((u2 ×
(power − alpha) + 1 − power)/(u1 × (power − alpha) + 1 − power))
}

##############FPR: FIGURE 4################################################
######varying SN 0.4 to 0.8 by 0.01####
SNv = seq(0.4, 0.8, by = 0.01)

powerv = rep(0, length(SNv))
for(i in 1:length(SNv)){
  powerv[i] = powercal(beta0, beta1, SNv[i], SP)
}

FNRv = matrix(0, ncol = 4, nrow = length(SNv))
taus = c(0.05, 0.5, 0.95)

for(i in 1:length(SNv)){
  for(j in 1:length(taus)){
    FNRv[i, j] = FNRfun(taus[j], ipH0.a, ipH0.b, alpha, powerv[i])
  }
  FNRv[i, j + 1] = FNRavg(ipH0.a, ipH0.b, alpha, powerv[i])
}

plot(SNv, FNRv[,2], cex = 0.5, xlab = “Sensitivity”, ylab = “FNR”, ylim = c(0, 1))
points(SNv, FNRv[,1], cex = 0.5, col = “blue”)
points(SNv, FNRv[,3], cex = 0.5, col = “red”)
points(SNv, FNRv[,4], cex = 0.5, col = “brown”, pch = 2)
legend(0.4, 1, c(“tau = 0.05”, “tau = 0.5”, “tau = 0.95”, “Mean”), col = c(“blue”, “black”, “red”,
“brown”), pch = c(rep(1, 3), 2))

##############FPR: FIGURE 6################################################
###varying lower limit from 0.4 to 0.8####
u1v = seq(0.4, 0.8, by = 0.01)

power = powercal(beta0, beta1, SN = 0.5, SP)

FNRv = matrix(0, ncol = 4, nrow = length(u1v))
taus = c(0.05, 0.5, 0.95)

for(i in 1:length(u1v)){
  for(j in 1:length(taus)){
    FNRv[i, j] = FNRfun(taus[j], u1v[i], ipH0.b, alpha, power)
```

```
  }
  FNRv[i, j + 1] = FNRavg(u1v[i], ipH0.b, alpha, power)
}
plot(u1v, FNRv[,2], cex = 0.5, xlab = “Lower limit of pi”, ylab = “FNR”, ylim = c(0, 1))
points(u1v, FNRv[,1], cex = 0.5, col = “blue”)
points(u1v, FNRv[,3], cex = 0.5, col = “red”)
points(u1v, FNRv[,4], cex = 0.5, col = “brown”, pch = 2)
legend(0.4, 1, c(“tau = 0.05”, “tau = 0.5”, “tau = 0.95”, “Mean”), col = c(“blue”, “black”, “red”,
“brown”), pch = c(rep(1, 3), 2))
```

Supplementary File 5: Theoretical Calculations of the Distributions of False Positive Rates (FPR) and False Negative Rates (FNR) while Varying Sensitivity (SN) and Specificity (SP) such that SN > SP—Sensitivity Analyses

```
###setting###
n < −3000   #size of planned study 1500 cases and 1500 controls
SN < −0.5 #sensitivity
SP < −0.9 #specificity
px1 < −0.3 #prevalence of exposure
alpha < −0.05 #critical p-value for hypothesis testing
##
beta0 < −0 #logit parameter of background rate of outcome/disease
beta1 < −log(1.6) #presumed true log-OR

####parameters of prior on null being true f(pi)~Uniform(ipH0.a, ipH0.b)
ipH0.a < −0.7 #lower bound PROBABILITY null is true
ipH0.b < −1.0 #upper bound PROBABILITY null is true

####Function to calculate the power with input beta0, beta1, SN, SP###
powercal = function(beta0, beta1, SN, SP){
   b0n < −SP × (1 − px1) × exp(beta0)/(1 + exp(beta0)) + (1 − SN) × px1 × exp(beta0 + beta1)/(1 +
exp(beta0 + beta1))
   b0d < −SP × (1 − px1)/(1 + exp(beta0)) + (1 − SN) × px1/(1 + exp(beta0 + beta1))
   b0 < −log(b0n/b0d)
   b1n < −(1 − SP) × (1 − px1) × exp(beta0)/(1 + exp(beta0)) + SN × px1 × exp(beta0 + beta1)/(1 +
exp(beta0 + beta1))
   b1d < −(1 − SP) × (1 − px1)/(1 + exp(beta0)) + SN × px1/(1 + exp(beta0 + beta1))
   b1 < −log(b1n/b1d) − b0

   V22 < −(1 + exp(b0))^2/(exp(b0) × (1 − px1)) + (1 + exp(b0 + b1))^2/(exp(b0 + b1) × px1)
   Z < −qnorm((1 − alpha/2), mean = 0, sd = 1, lower.tail = TRUE, log.p = FALSE)
   q1 = −Z + sqrt(n) × b1/sqrt(V22)
   q2 = −Z − sqrt(n) × b1/sqrt(V22)
   power = pnorm(q1) + pnorm(q2)
```

```
    return(power)

}
##############################################################################
##############FPR###############################################################
##############################################################################

####funcion to calculate the tau-th quantile of FPR###
####power can be calculated by powercal function first###

FPRfun = function(tau, u1, u2, alpha, power){
  (tau × (u2 − u1) + u1) × alpha/(power − (tau × (u2 − u1) + u1) × (power − alpha))
}

##function to calculate the expectation of FPR###
FPRavg = function(u1, u2, alpha, power){
   alpha/(alpha − power) − alpha × power/(u2 − u1)/((alpha − power)^2) × log((u2 × (alpha − power) +
power)/(u1 × (alpha − power) + power))
}

##########################################FIGURE 7

######varying SN 0.6 to 0.9 by 0.01####
####SP fixed at 0.5
SP < −0.5
SNv = seq(0.6, 0.9, by = 0.01)

powerv = rep(0, length(SNv))
for(i in 1:length(SNv)){
  powerv[i] = powercal(beta0, beta1, SNv[i], SP)
}

FPRv = matrix(0, ncol = 4, nrow = length(SNv))
taus = c(0.05, 0.5, 0.95)

for(i in 1:length(SNv)){
  for(j in 1:length(taus)){
    FPRv[i, j] = FPRfun(taus[j], ipH0.a, ipH0.b, alpha, powerv[i])
  }
  FPRv[i, j + 1] = FPRavg(ipH0.a, ipH0.b, alpha, powerv[i])
}

plot(SNv, FPRv[,2], cex = 0.5, xlab = “Sensitivity”, ylab = “FPR”, ylim = c(0, 1))
points(SNv, FPRv[,1], cex = 0.5, col = “blue”)
```

```
points(SNv, FPRv[,3], cex = 0.5, col = “red”)
points(SNv, FPRv[,4], cex = 0.5, col = “brown”, pch = 2)
legend(0.7, 1, c(“tau = 0.05”, “tau = 0.5”, “tau = 0.95”, “Mean”), col = c(“blue”, “black”, “red”,
“brown”), pch = c(rep(1, 3), 2))

########################################################################
##############FNR#######################################################
########################################################################

#####Function to calculate the tau-th quantile of FNR######

FNRfun = function(tau, u1, u2, alpha, power){
  (1 − power) ×(1 − (u2 − tau ×(u2 − u1)))/(1 – power + (u2 − tau ×(u2 − u1)) ×(power − alpha))
}

###Function to calculate the expectation of FNR####

FNRavg = function(u1, u2, alpha, power){

  (1 − power)/(alpha − power) + (1 − power) ×(1 − alpha)/(u2 − u1)/((alpha − power)^2) ×log((u2 ×
(power − alpha) + 1 − power)/(u1 ×(power − alpha) + 1 − power))
}

########################################FIGURE 8
######varying SN 0.6 to 0.9 by 0.01####
####SP fixed at 0.5
SP < −0.5
SNv = seq(0.6, 0.9, by = 0.01)

powerv = rep(0, length(SNv))
for(i in 1:length(SNv)){
  powerv[i] = powercal(beta0, beta1, SNv[i], SP)
}

FNRv = matrix(0, ncol = 4, nrow = length(SNv))
taus = c(0.05, 0.5, 0.95)

for(i in 1:length(SNv)){
  for(j in 1:length(taus)){
    FNRv[i, j] = FNRfun(taus[j], ipH0.a, ipH0.b, alpha, powerv[i])
  }
  FNRv[i, j + 1] = FNRavg(ipH0.a, ipH0.b, alpha, powerv[i])
}

plot(SNv, FNRv[,2], cex = 0.5, xlab = “Sensitivity”, ylab = “FNR”, ylim = c(0, 1))
```

```
points(SNv, FNRv[,1], cex = 0.5, col = “blue”)
points(SNv, FNRv[,3], cex = 0.5, col = “red”)
points(SNv, FNRv[,4], cex = 0.5, col = “brown”, pch = 2)
legend(0.7, 1, c(“tau = 0.05”, “tau = 0.5”, “tau = 0.95”, “Mean”), col = c(“blue”, “black”, “red”,
“brown”), pch = c(rep(1, 3), 2))
```

© 2014 by the authors; licensee MDPI, Basel, Switzerland. This article is an open access article distributed under the terms and conditions of the Creative Commons Attribution license (http://creativecommons.org/licenses/by/4.0/).
